# The timing of TGF-β inhibition affects the generation of antigen-specific CD8^+^ T Cells

**DOI:** 10.1186/1471-2172-14-30

**Published:** 2013-07-17

**Authors:** Jon G Quatromoni, Eiji Suzuki, Olugbenga Okusanya, Brendan F Judy, Pratik Bhojnagarwala, Ollin Venegas, Evgeniy Eruslanov, Jarrod D Predina, Steven M Albelda, Sunil Singhal

**Affiliations:** 1 Division of Thoracic Surgery, Perelman School of Medicine at the University of Pennsylvania, 6 White Building, 3400 Spruce Street, Philadelphia, PA 19104, USA; 2 Department of Surgery, Perelman School of Medicine at the University of Pennsylvania, 3400 Spruce Street, Philadelphia, PA 19104, USA; 3 Division of Pulmonary, Allergy and Critical Care Medicine, Department of Medicine, Perelman School of Medicine at the University of Pennsylvania, 1015F ARC, 3615 Civic Center Blvd., Philadelphia, PA 19104, USA; 4 Department of Medicine, Perelman School of Medicine at the University of Pennsylvania, 3400 Spruce Street, Philadelphia, PA 19104, USA

**Keywords:** Malignant mesothelioma, Tumor immunology, Immune suppression, TGF-β, CD8^+^ Cytotoxic T cell

## Abstract

**Background:**

Transforming growth factor (TGF)-β is a potent immunosuppressive cytokine necessary for cancer growth. Animal and human studies have shown that pharmacologic inhibition of TGF-β slows the growth rate of established tumors and occasionally eradicates them altogether. We observed, paradoxically, that inhibiting TGF-β before exposing animals to tumor cells increases tumor growth kinetics. We hypothesized that TGF-β is necessary for the anti-tumor effects of cytotoxic CD8^+^ T lymphocytes (CTLs) during the early stages of tumor initiation.

**Methods:**

BALB/c mice were pretreated with a blocking soluble TGF-β receptor (sTGF-βR, TGF-β-blockade group, n=20) or IgG2a (Control group, n=20) before tumor inoculation. Tumor size was followed for 6 weeks. *In vivo* lymphocyte assays and depletion experiments were then performed to investigate the immunological basis of our results. Lastly, animals were pretreated with either sTGF-βR (n=6) or IgG2a (n=6) prior to immunization with an adenoviral vector encoding the human papillomavirus E7 gene (Ad.E7). One week later, flow cytometry was utilized to measure the number of splenic E7-specific CD8^+^ T cells.

**Results:**

Inhibition of TGF-β before the injection of tumor cells resulted in significantly larger average tumor volumes on days 11, 17, 22, 26 and 32 post tumor-inoculation (p < 0.05). This effect was due to the inhibition of CTLs, as it was not present in mice with severe combined immunodeficiency (SCID) or those depleted of CD8^+^ T cells. Furthermore, pretreatment with sTGF-βR inhibited tumor-specific CTL activity in a Winn Assay. Tumors grew to a much larger size when mixed with CD8^+^ T cells from mice pretreated with sTGF-βR than when mixed with CD8^+^ T cells from mice in the control group: 96 mm^3^ vs. 22.5 mm^3^, respectively (p < 0.05). In addition, fewer CD8^+^ T cells were generated in Ad.E7-immunized mice pretreated with sTGF-βR than in mice from the control group: 0.6% total CD8^+^ T cells vs. 1.9%, respectively (p < 0.05).

**Conclusions:**

These studies provide the first *in vivo* evidence that TGF-β may be necessary for anti-tumor immune responses in certain cancers. This finding has important implications for our understanding of anti-tumor immune responses, the role of TGF-β in the immune system, and the future development of TGF-β inhibiting drugs.

## Background

Transforming growth factor (TGF)-β is a multifunctional cytokine that is capable of either stimulating or inhibiting growth and differentiation of a wide range of cell types, including many of those in the immune system [1-3]. Understanding the role of TGF-β in tumor biology is important to both basic science and translational medicine [4-6].

TGF-β functions primarily as an immunosuppressive cytokine in the tumor microenvironment due to its ability to interfere with the generation, expansion, and function of anti-tumor immune cells [2-4,7]. In a number of *in vitro* and *ex vivo* studies, TGF-β has been associated with the suppression of growth and/or activity of T cells [8-12], NK cells [6,13], and dendritic cells [14-16]. The current *in vivo* evidence further supports this hypothesis; using a number of approaches that include anti-TGF-β antibodies, soluble receptors, or TGF-β-binding proteins [6,17], translational investigators have consistently reported that the blockade of TGF-β is therapeutically useful in a number of murine tumor systems, including renal cell cancer [18], melanoma [19], hepatocellular carcinoma [20], and glioma [21].

**Figure 1:**
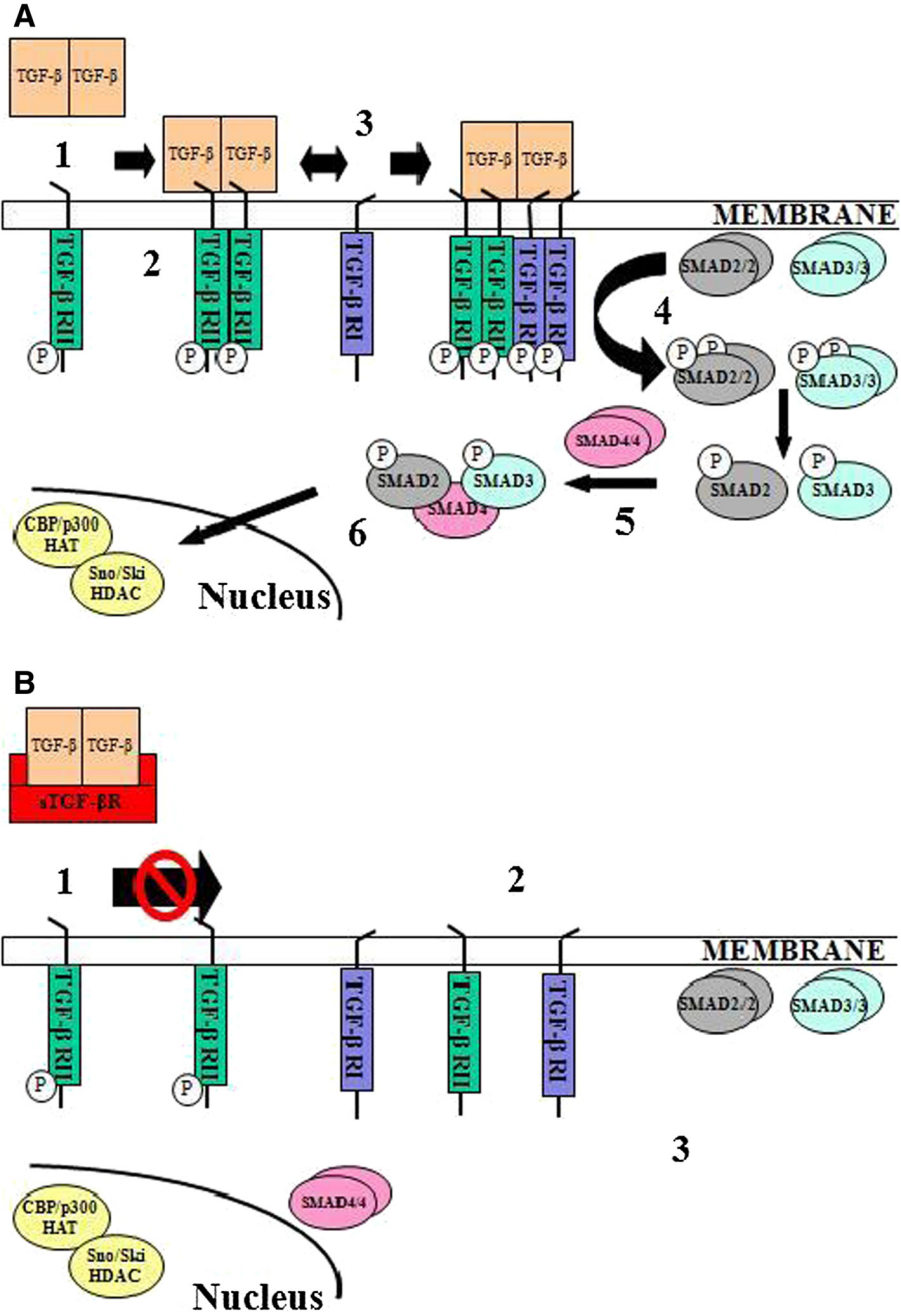
**TGF-β signaling pathway.** Panel **A**: unimpeded TGF-β signaling pathway: 1. Ligand binding induces TGF-β receptor type II (TGF-βRII) dimerization. 2. TGF-β type I (TGF-βRI) receptor recruitment. 3. TGF-βRI phosphorylation and activation. 4. SMAD phosphorylation by TGF-βRI. 5. Co-SMAD4 binding. 7. Translocation to the nucleus to activate or repress target genes. Panel **B**: sTGF-βR-mediated inhibition of TGF-β signaling. 1. Ligand does not bind TGF-βRII. 2. TGF-βRI is not recruited, phosphorylated, and activated. 3. Downstream effect is inhibition of TGF-βRII mediated phosphorylation of SMAD proteins.

Our group previously reported similar anti-tumor effects after administering a soluble type II TGF-β receptor (sTGF-βR) that binds and neutralizes TGF-β1 and TGF-β3 in a murine model of established mesothelioma tumors (Figure 1). In that study, we found that tumor inhibition by sTGF-βR was due to enhanced activity of anti-tumor cytotoxic CD8^+^ T lymphocytes (CTLs) [22]. In an attempt to augment the anti-tumor effects of TGF-β-blockade, we also administered sTGF-βR to mice prior to the injection of various cancer cell lines, including the mesothelioma cell line AB12. We observed, paradoxically, that the administration of sTGF-βR prior to injection of cancer cells resulted in an increased growth rate of AB12 tumors.

The purpose of this study is to further characterize the role of TGF-β-inhibition in tumorigenesis. The findings of these studies have important implications for our overall understanding of the generation of anti-tumor immune responses, the role of TGF-β in the immune system, and the future use and development of drugs that inhibit TGF-β.

## Methods

### Study animals

Pathogen-free female BALB/c and C57BL/6 mice (6–8 weeks old; weight ~20-25 g) were purchased from Taconic Labs (Germantown, NY). CB-17 SCID mice (6–8 weeks old; weight ~20-25 g) were bred at the Wistar Institute (Philadelphia, PA). All mice were maintained in a pathogen-free animal facility for at least 1 week before each experiment. The animal use committees of the Wistar Institute and University of Pennsylvania approved all protocols in compliance with the Guide for the Care and Use of Laboratory Animals.

### Cell lines

Four murine tumor cell lines were investigated in this study: the AB12 and AB-1 mesothelioma cell lines, the TC-1 non-small-cell lung carcinoma cell line, and the L1C2 bronchoalveolar carcinoma cell line. The non-malignant mink lung epithelial cells (MLECs) were also investigated. The AB12 and AB-1 cell lines were obtained from Dr. Bruce Robinson [23]. These lines were derived in BALB/c mice and grow well as flank tumors in this model [24]. The ability of these lines to secrete TGF-β spontaneously in culture has been studied in detail [25]. AB12 cells secrete large amounts of TGF-β (462 pmol/10^6^ cells/24 hours), mostly in its latent form. AB-1 cells, on the other hand, do not secrete significant quantities of TGF-β [25]. The TC-1 cell line was generated by transduction of C57BL/6 primary lung epithelial cells with a retroviral vector expressing HPV16 E6/E7 plus a retrovirus expressing activated c-Ha-*ras*[26]. This line is highly tumorigenic in C57BL/6 mice and grows well as flank tumors in this model. The L1C2 cell line, obtained from the American Type Culture Collection (Rockville, MD), is highly tumorigenic in BALB/c mice and grows well as flank tumors in this model. MLECs, previously transfected with a plasminogen activator inhibitor-1 (PAI-1) promoter-luciferase construct, were obtained from Dr. Daniel Rifkin [27].

AB12, AB-1, L1C2, and MLECs were cultured and maintained in high glucose Dulbecco’s modified Eagle’s medium (DMEM; Mediatech, Washington DC) supplemented with 10% fetal bovine serum (FBS; Georgia Biotechnology, Atlanta GA), 100 units/mL penicillin, 100 μg/mL streptomycin, and 2 mM glutamine. TC-1 was cultured in in RPMI 1640 (Life Technologies, Inc.) supplemented with 10% FBS, 100 units/mL penicillin, 100 μg/mL streptomycin, and 2 mM glutamine. All cell lines were regularly tested and maintained negative for *Mycoplasma* species.

### Quantitative TGF-β bioassay

TGF-β production by the tumor cell lines was quantified using a highly sensitive and specific, nonradioactive, bioassay [27]. This bioassay is based on the ability of TGF-β to induce PAI-1 expression. Briefly, MLECs stably transfected with a construct containing the human PAI-1 promoter fused to the firefly luciferase reporter gene were suspended in DMEM containing 10% FBS and seeded in 96-well plates at a density of 1.6×10^4^ cells per well. Samples and standards (human platelet-derived TGF-β1 protein; BD Biosciences PharMingen, San Diego, CA) were added in triplicate to the plate of MLECs and incubated for 16 hours at 37°C in a 5% CO_2_ incubator. Cells were then lysed with 1x cell-lysis buffer (Analytical Luminescence, Promega, Madison, WI) and the lysates were transferred to a 96-well plate. Both substrate A and substrate B (Analytical Luminescence, Promega) were then added to the samples. Luciferase activity (a proxy for quantity of TGF-β in the sample) was measured using an ML1000 luminometer (Dynatech Laboratories Inc., Alexandria, VA) and reported as relative light units (RLU).

### Soluble TGF-β inhibitor

The soluble recombinant murine TGF-β type II-murine Fc: IgG2a chimeric protein (sTGF-βR) has previously been described [22,28]. This chimeric protein binds and inhibits TGF-β1 and TGF-β3 in the 1 nM range and has a half-life in mouse plasma of 14 days. Previous studies have shown biological effects at 1 mg/kg [28], 2 mg/kg [29], and 5 mg/kg [30]. Based on these reports, we injected sTGF-βR at a concentration of 1.0 mg/kg in all of our experiments. Murine IgG2a antibody was used as a control and injected at the same concentration. The use of murine IgG2a as a control has been described in previous studies [29,31].

### Animal tumor models

To confirm the effect of sTGF-βR on established tumors [22], we injected BALB/c mice in 1 flank with 1×10^6^ AB12 tumor cells and then initiated treatment with sTGF-βR (TGF-β-blockade group, n=5) or mouse IgG2a (Control group, n=5) when the tumors reached a minimal volume of 100 mm^3^ (approximately 10 days after tumor cell-inoculation). Animals in the TGF-β-blockade group received 1 intraperitoneal (IP) injection of sTGF-βR, once every 3 days, for a total of 6 doses (a 16-day course). Control animals received murine IgG2a according to the same schedule. We then followed tumor burden with serial estimates of tumor volume.

To test the efficacy of pretreatment with sTGF-βR, we administered sTGF-βR or IgG2a 2 days before inoculation of 1×10^6^ AB12, AB-1, L1C2, or TC-1 tumor cells into the flank of each animal. The TGF-β-blockade group received 1 IP injection of sTGF-βR, once every 3 days, for a total of 3 doses (a 7-day course). The control group received murine IgG2a according to the same schedule. We then followed tumor burden with serial estimates of tumor volume. As part of our investigation into the basis of our results, this protocol was subsequently implemented in SCID animals using AB12 cells.

Lastly, we created a reproducible animal model of metastatic disease to study sTGF-βR in this context. First, we injected 1×10^6^ AB12 tumor cells into the right flank of animals. When the tumors reached a minimal volume of 100 mm^3^, we initiated treatment with sTGF-βR or IgG2a: animals received 1 injection, once every 3 days. After 3 doses of either sTGF-βR or IgG2a, 1×10^6^ AB12 cells were inoculated into the opposite flank, thus modeling a “metastatic focus”. After tumor re-challenge, 3 additional doses of sTGF-βR or IgG2a were administered. We then followed tumor burden in the primary and secondary inoculation sites with serial estimates of tumor volume.

In all instances, tumor volume was calculated according to the formula (π*long axis*short axis*short axis)/6, as described previously [22]. We measured tumor volume at least twice weekly. Unless otherwise mentioned, each control or experimental group had a minimum of 5 mice. Each experiment was repeated at least once.

### Flow cytometry on tumor-infiltrating lymphocytes and lymphocytes in the tumor-draining lymph nodes

To study tumor-infiltrating lymphocytes (TILs) and lymphocytes in the tumor-draining lymph nodes (TDLNs), we compared 3 groups: 1 non tumor-bearing group (Naïve group, n=9) and 2 groups of tumor-bearing animals (Control group, n=9 and TGF-β-blockade group, n=9). The naïve group consisted of BALB/c mice that received a one-time IP injection of BD Matrigel™ matrix (BD Biosciences, Bedford, MA) without tumor cells into both flanks. The control group consisted of BALB/c mice that were injected with 1x10^6^ AB12 cells in 250 μL of serum-free DMEM media mixed with 250 μL of BD Matrigel™ matrix into both flanks. Two days before tumor cell-inoculation and once every 3 days thereafter, for a total of 3 doses, these mice received IP injections of IgG2a (Day −2, Day 1, and Day 4). The TGF-β-blockade group consisted of BALB/c mice that were injected with 1×10^6^ AB12 cells in 250 μL of serum free DMEM media mixed with 250 μL of BD Matrigel™ matrix into both flanks. Two days before tumor cell-inoculation and once every 3 days thereafter, for a total of 3 doses, these mice received IP injections of sTGF-βR (Day −2, Day 1, and Day 4).

Two, 4, and 7 days after tumor cell-inoculation, tumors and bilateral inguinal lymph nodes from both the control (n=3) and TGF-β-blockade (n=3) groups were harvested. Single-cell suspensions were generated by mincing these tissues on ice and subsequently filtering them through a 70μm BD Falcon™ cell strainer (BD Biosciences Discovery Labware, Bedford, MA). These populations were then stained with the following antibodies: allophycocyanin (APC) conjugated to rat anti-mouse CD45 or CD8a (BD Biosciences PharMingen, San Diego, CA), fluorescein isothiocyanate (FITC) conjugated to rat anti-mouse CD4, CD11c (BD Biosciences PharMingen, San Diego, CA), or MHC class I (34-1-2s; eBioscience, San Diego, CA), and phycoerythrin (PE) conjugated to rat anti-mouse CD8a, CD11c, CD86 (BD Biosciences PharMingen, San Diego, CA), or MHC class II (M5/114.15.2; eBioscience). We then used flow cytometry to analyze these populations (FACScalibur, Becton-Dickinson, Mountain View, CA).

Of note, the rationale for inoculation of AB12 tumor cells in a Matrigel™ matrix for this experiment was based on the difficulty of generating single cells suspensions from 2-day old tumors.

### Animal vaccine models

To determine if TGF-β-inhibition affects the ability of mice to generate antigen-specific CD8^+^ T cells, we studied the effect of pretreatment with sTGF-βR in animals immunized against the human papillomavirus E7 protein using an adenoviral vaccine (Ad.E7). First, 6-to-8 week-old female C57BL/6 animals were treated with either sTGF-βR or IgG2a. Two days later, these animals were immunized with Ad.E7 via subcutaneous injection of 1×10^9^ plaque-forming units (pfu), as previously described [32,33]. Seven days after immunization, splenocytes were isolated from each group and analyzed by flow cytometry to establish the percentage of E7-specific CD8^+^ T cells (as determined by tetramer binding).

To determine if TGF-β-inhibition affects the period of viability of *established* antigen-specific CD8^+^ T cells, 6-to-8 week-old female C57BL/6 mice were immunized with 1×10^9^ pfu of Ad.E7 and treated 7 days later with either sTGF-βR or IgG2a. Then, 7 days after treatment (14 days after immunization), splenocytes from each group (n=3) were analyzed by flow cytometry to establish the percentage of E7-specific CD8^+^ T cells (as determined by tetramer binding).

Unless otherwise mentioned, each control group or experimental group had a minimum of 3 mice. Each experiment was repeated at least once.

### Analysis of E7-specific CD8^+^ T cells by flow cytometry

Tetramer staining of spleen cells was performed as previously described [34]. Single cell suspensions were generated by filtering spleens through a 70 μm BD Falcon™ cell strainer and then incubating the isolated cells for 15 minutes with BD PharM Lyse™, an ammonium chloride-based red blood cell-lysing reagent (BD Biosciences PharMingen, San Diego, CA). The remaining viable cells were incubated with anti-CD16 mAb (eBioscience, San Diego, CA) for 30 minutes to block non-specific binding of spleen cells to the Fc portion of test-antibody. Then, the spleen cells were stained FITC-conjugated anti-CD8a antibody (BD Biosciences PharMingen) and APC-conjugated E7 tetramer for 30 minutes and 1.5 hours, respectively.

APC-labeled H-2D^b^ tetramers loaded with E7 peptide (RAHYNIVTF) were obtained from the National Institute of Allergy and Infectious Diseases (eNIAID, Bethesda, MD) tetramer core. Flow cytometry was performed using a DakoCytomation CyAn (DakoCytomation, Fort Collins, CO).

### *In Vivo* depletion of CD8^+^ T cells

To deplete CD8^+^ T cells prior to, and during, treatments with sTGF-βR or IgG2a in our AB12 tumor model, mice received 200 μg IP injections of monoclonal antibody purified from the anti-CD8 hybridoma 53–6.7 (obtained from the American Type Culture Collection). Mice received injections both 1 and 3 days prior to inoculation with AB12 tumor cells. Thereafter, a maintenance dose was administered once every 7 days throughout the experimental period to ensure continued depletion. CD8^+^ T cell depletion was confirmed by flow cytometric analysis of spleen cells (preparation described above) at the time of tumor injection and weekly thereafter.

### Evaluation of effector function (Winn Assay)

We performed Winn Assays as previously described [35]. This assay allows for assessment of anti-tumor activity of immune effector cells *in vivo* without the need for *ex vivo* stimulation. We first prepared a single-cell suspension of splenocytes as described above. Then, CD8^+^ T cells were isolated from this suspension using the MACs system (Miltenyi Biotec, Auburn, CA). This cell population contained greater than 90% CD8^+^ T cells as determined by flow cytometry (data not shown). The CD8^+^ T cell-enriched populations from non tumor-bearing (naïve), IgG2a-pretreated animals (control), or sTGF-βR-pretreated animals (TGF-β-blockade) were admixed with viable AB12 tumor cells at a ratio of 3 purified CD8^+^ T cells per 1 tumor cell. This ratio has previously been determined to be optimal for detecting positive and negative effects [36]. This mixture was then inoculated subcutaneously (SC) into the flanks of naïve BALB/c mice. Each mouse thus received a total of 0.5×10^6^ tumor cells and 1.5×10^6^ CD8^+^ T cells. Tumor growth was measured after 1 week and expressed as the mean ± standard error of the mean. Each group contained at least 5 mice unless otherwise stated.

### Statistical analysis

We implemented unpaired Student’s t-tests to compare differences in continuous variables between control and experimental groups. Analysis of variance (ANOVA) with post-hoc testing was used for multiple comparisons. We considered differences statistically significant when the p-value was less than 0.05. Statistical analysis was conducted using the StatView 5.0 for Windows program (Abacus Concepts Inc., Berkeley, CA).

## Results

### AB12 and TC-1 cells produce a large amount of TGF-β

To determine the level of TGF-β production by the murine cancer cell lines under investigation, we measured soluble TGF-β by the quantitative bioassay described above. AB12 and TC-1 cell lines produced more TGF-β (277.3 pg/mL and 263.3 pg/mL, respectively) than AB-1 and L1C2 (88.9 pg/mL and 92.3 pg/mL, respectively).

### The administration of sTGF-βR to animals with established AB12 tumors inhibits tumor growth, while treatment before AB12 inoculation stimulates tumor growth

**Figure 2:**
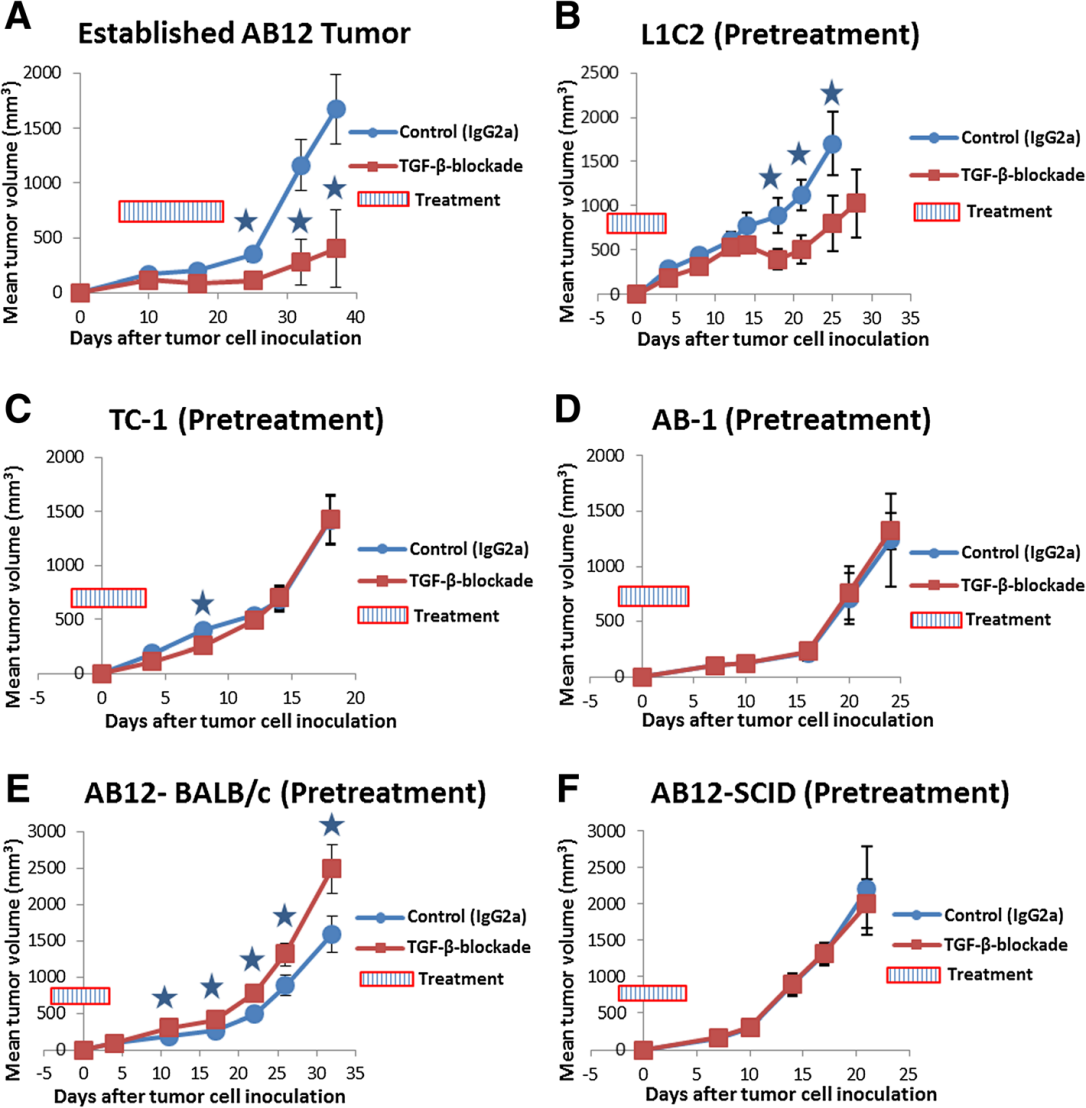
**The effects of sTGF-βR on tumor growth according to timing of administration relative to tumor-challenge, animal model, and murine lung cancer cell line.** In all graphs, tumor volume was calculated by the formula (π*long axis*short axis*short axis)/6 and displayed as mean volume (mm^3^). Error bars represent standard error of the mean (SEM). IgG2a (control) and sTGF-βR were administered according to specific schedules. Animals with small (150 mm^3^), established tumors (Graph A) received IP injections of IgG2a (control) or sTGF-βR, once every 3 days, for a total of 6 doses. Pretreatment animals (Graphs B-F) received IP injections of IgG2a (control) or sTGF-βR two days before tumor cell-inoculation, and once every 3 days thereafter, for a total of 3 doses. (**A**). AB12 (established tumors), BALB/c animals (n=5). Established AB12 tumors in animals treated with sTGF-βR were significantly smaller than tumors in control animals treated with IgG2a at multiple time points (* = p < 0.05). (**B**). L1C2 (pretreatment), BALB/c animals (n=5). L1C2 tumors in animals pretreated with sTGF-βR were significantly smaller than tumor in control animals pretreated with IgG2a at multiple time points (* = p < 0.05). (**C**). TC-1 (pretreatment), C57BL/6 animals (n=5). TC-1 tumors in animals pretreated with sTGF-βR were significantly smaller than tumors in control animals pretreated with IgG2a at one time point (* = p < 0.05). (**D**). AB-1 (pretreatment), BALB/c animals (n=5). No significant difference in AB-1 tumor size was evident between control animals pretreated with IgG2a and animals pretreated with sTGF-βR (p > 0.05). (**E**). AB12 (pretreatment), BALB/c animals (n=5). AB12 tumors in animals pretreated with sTGF-βR were significantly larger than tumors in control animals pretreated with IgG2a at multiple time points (* = p < 0.05). (**F**). AB12 (pretreatment), SCID animals (n=5). No significant difference in AB12 tumor size was evident between control animals that received IgG2a and animals that received sTGF-βR (p > 0.05).

**Figure 3:**
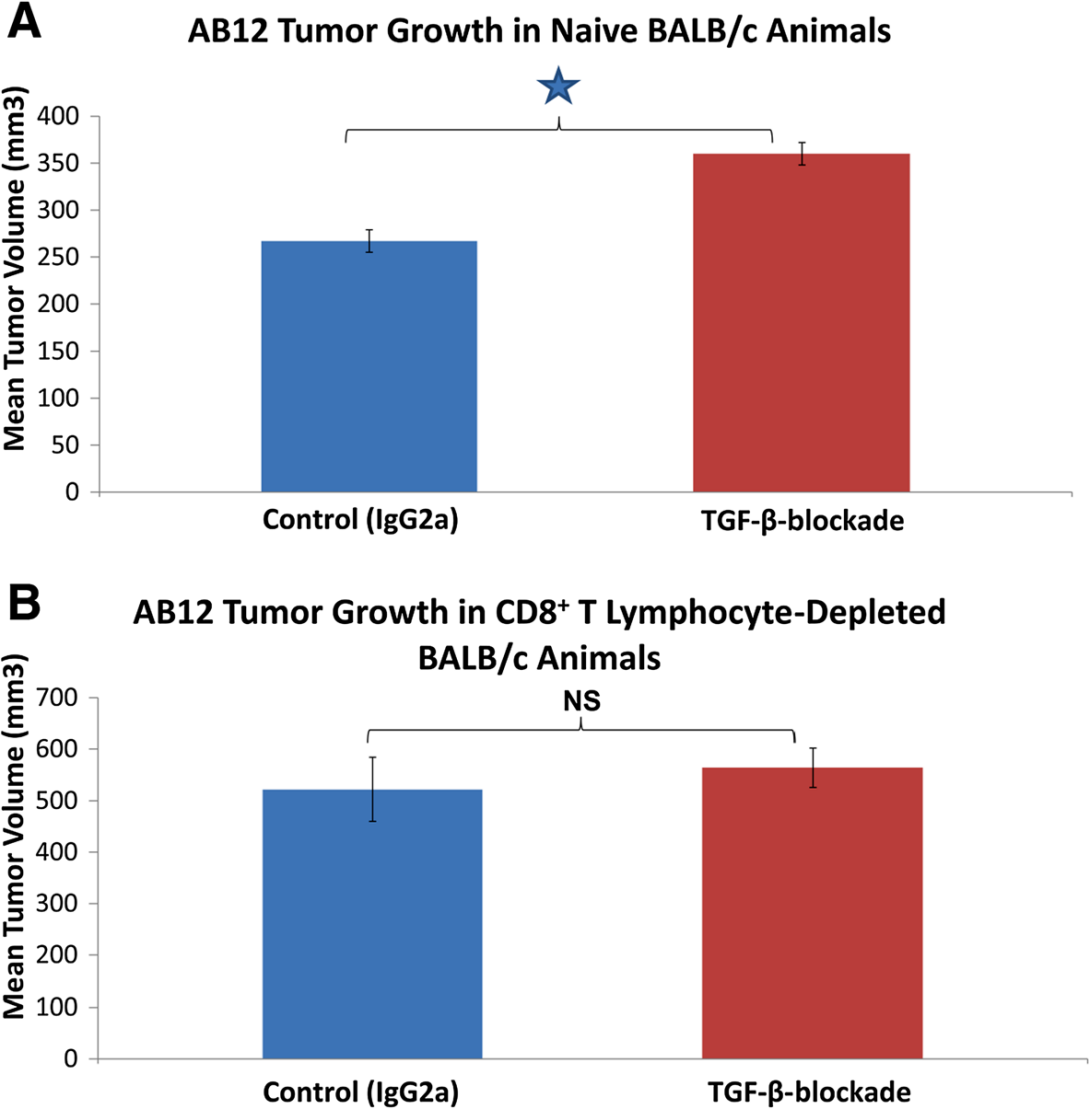
**The effect of pretreatment with sTGF-βR on AB12 tumor growth in CD8**^**+ **^**T cell-depleted animals.** In all graphs, tumor volume was calculated by the formula (π*long axis*short axis*short axis)/6 and displayed as mean volume (mm^3^). Error bars represent standard error of the mean (SEM). (**A**). Intact, non CD8^+^ T cell-depleted animals. Two days before tumor cell-inoculation and once every 3 days thereafter (for a total of 3 doses), groups of mice (n=5) received IP injections of IgG2a (control) or sTGF-βR (TGF-β-blockade). Tumor volume at 17 days post tumor cell-inoculation is shown. Tumors in mice pretreated with sTGF-βR were significantly larger than tumors in control animals pretreated with IgG2a (* = p < 0.05). (**B**). CD8^+^ T cell-depleted animals. Two days before tumor cell-inoculation and once every 3 days thereafter (for a total of 3 doses), groups of CD8^+^ T cell-depleted mice (n=5) received IP injections of IgG2a (control) or sTGF-βR. Tumor volume at 17 days post-tumor cell inoculation is shown. Depletion of CD8^+^ T cells resulted in larger absolute tumors in both groups. No significant difference in tumor size was evident between tumors of control animals pretreated with IgG2a and animals pretreated with sTGF-βR (NS = p > 0.05).

Previous studies have shown that the administration of sTGF-βR significantly decreases the growth of established AB12 tumors [22]. We conducted a similar experiment to confirm these findings. As expected, the administration of sTGF-βR into mice with established AB12 tumors resulted in significantly smaller tumors compared to control animals receiving IgG2a on days 25 (110 mm^3^ vs. 345 mm^3^), 32 (275 mm^3^ vs. 1165 mm^3^), and 37 (400 mm^3^ vs. 1675 mm^3^) post tumor-inoculation (p < 0.05) (Figure 2A). However, the pretreatment of animals with sTGF-βR, before AB12 inoculation, resulted in increased tumor growth at multiple time points compared to control animals: AB12 tumors were significantly larger on days 11 (305 mm^3^ vs. 185 mm^3^), 17 (410 mm^3^ vs. 265 mm^3^), 22 (780 mm^3^ vs. 495 mm^3^), 26 (1325 mm^3^ vs. 880 mm^3^), and 32 (2500 mm^3^ vs. 1595 mm^3^) post tumor-inoculation (p < 0.05) (Figure 2E). In contrast, the pretreatment of animals with sTGF-βR before L1C2 or TC-1 inoculation inhibited tumor growth compared to control animals (Figure 2B and 2C). Pretreatment with sTGF-βR before AB1 inoculation had no effect on tumor growth (Figure 2D). This experiment was repeated more than 3 times with similar results (Figure 3A).

### The increased rate of AB12 tumor growth after pretreatment with sTGF-βR is abolished in the SCID animal model

Previous reports have suggested that TGF-β acts as a direct growth-inhibitor of certain cancer cell lines [37,38]. Neutralization of TGF-β might therefore induce more rapid growth. However, our lab has shown that TGF-β-inhibition results in neither direct stimulation nor inhibition of AB12 cell proliferation *in vitro*[22]. To assess the possibility of indirect immunologically-mediated effects of TGF-β on tumor cell growth, we repeated our pretreatment studies using the AB12 cell line in the immunodeficient CB-17 SCID animal model (which lacks B and T lymphocytes). The pretreatment of SCID mice with sTGF-βR before AB12 inoculation abolished the augmentation of growth seen in BALB/c mice (Figure 2F), as tumor growth rates did not differ between mice pretreated with sTGF-βR and control mice pretreated with IgG2a. These experiments show that the increased rate of tumor growth resulting from pretreatment with sTGF-βR in the BALB/c tumor model is not the result of neutralizing direct growth-inhibiting effects of TGF-β; rather, these results support an immunologically-mediated mechanism that is dependent on the presence of B and/or T cells.

### The increased rate of AB12 tumor growth after pretreatment with sTGF-βR is abolished in CD8^+^ T cell-depleted animals

We then designed a lymphocyte-depletion experiment to further probe the immunologic basis of our findings and determine which cells were responsible for this effect. We depleted CD8^+^ T cells after finding small numbers of CD4^+^ T cells in AB12 tumors by flow cytometry (data not shown). The pretreatment of naïve (non CD8^+^ T cell-depleted) BALB/c animals with sTGF-βR resulted in larger tumors compared to control animals pretreated with IgG2a (Figure 3A). At day 17, tumors in control mice were 260 mm^3^ compared to 350 mm^3^ in animals pretreated with sTGF-βR, a 34% augmentation of size (p < 0.05). However, when BALB/c mice were depleted of their CD8^+^ T cells, this significant difference in tumor growth rates between animals pretreated with sTGF-βR or IgG2a disappeared (Figure 3B). Mean tumor volume at day 17 in the animals pretreated with sTGF-βR was 550 mm^3^ compared to 520 mm^3^ in the control animals. This 5% difference in tumor growth was not statistically significant (p>0.05). These results, in combination with the SCID animal experiments, demonstrate that the stimulatory effect on tumor growth resulting from pretreatment with sTGF-βR relies on the presence of CD8^+^ T lymphocytes.

### Pretreatment with sTGF-βR before AB12 tumor challenge abolished tumor-specific CTL activity

The more rapid absolute growth of AB12 tumors in SCID and CD8^+^ T cell-depleted mice regardless of treatment (Figure 2) suggests that the wild-type BALB/c animals mount a tumor-specific, although ultimately ineffective, CD8^+^ T cell response against the tumor at early time points. We have previously documented the presence of anti-tumor CTLs that arise early in the course of tumor growth and then disappear as the tumors grow to larger sizes (>500 mm^3^) using an *in vivo* tumor neutralization assay (Winn Assay) [22,39]. In order to determine if the increased rate of AB12 tumor growth associated with sTGF-βR pretreatment was dependent on the inhibition of naturally-occurring endogenous anti-tumor CTL, we conducted a Winn Assay as outlined above.

**Figure 4:**
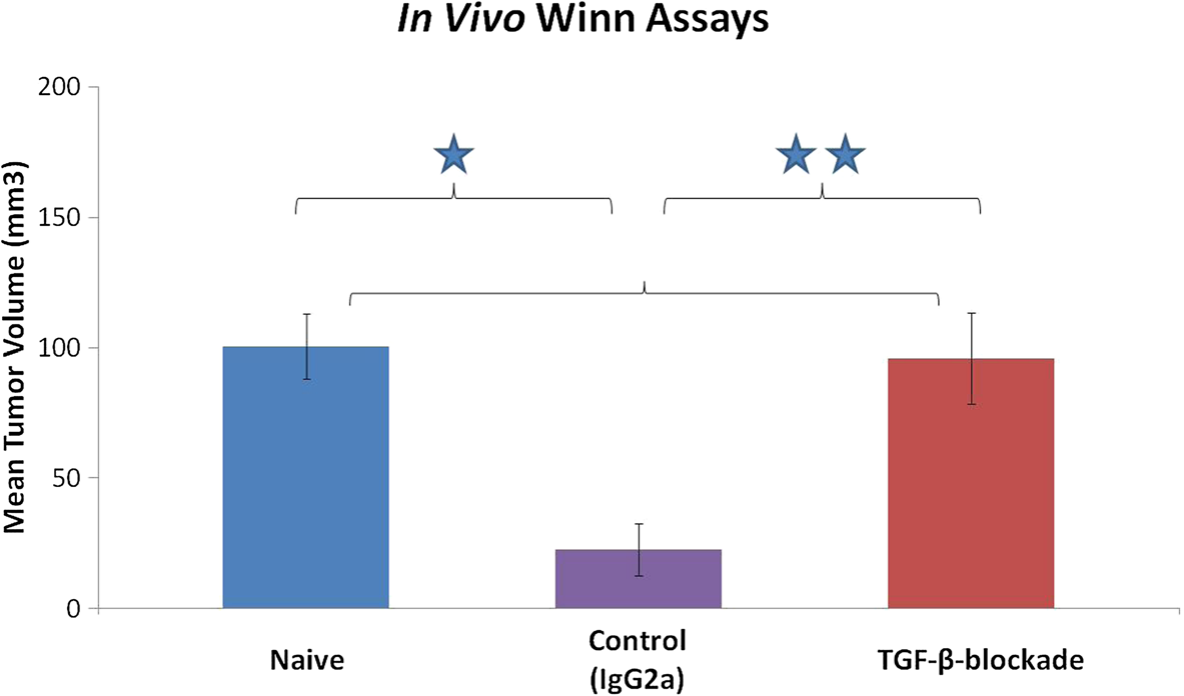
**Winn Assays to assess the *in vivo *activity of anti-tumor CD8**^**+ **^**T cells from animals treated with IgG2a (control) or sTGF-βR.** Tumor volume was calculated by the formula (π*long axis*short axis*short axis)/6 and displayed as mean volume (mm^3^). Error bars represent standard error of the mean (SEM). CD8^+^ T cells were isolated from the spleens of non tumor-bearing animals, tumor-bearing animals treated with IgG2a, or tumor-bearing animals treated with sTGF-βR. These CD8^+^ T cells were then mixed with fresh AB12 tumor cells according to a ratio of 3 CD8^+^ T cells to 1 AB12 tumor cell. These mixtures were then injected into the flanks of naïve BALB/c mice (n=5 per group). Tumors were then allowed to grow for 1 week. Tumors from mixtures of AB12 tumor cells and CD8^+^ T cells from non tumor-bearing animals grew to an average size of approximately 100 mm^3^ (“naïve” group). Tumors from mixtures of AB12 tumor cells and CD8^+^ T cells from animals treated with IgG2a grew to an average size of approximately 30 mm^3^ (“control” group), representing a 68% inhibition of growth (* = p < 0.01). However, tumors from mixtures of AB12 cells and CD8^+^ T cells from animals treated with sTGF-βR (“TGF-β-blockade” group) grew to a significantly larger average size than the control group (** = p < 0.05) and to the same degree as the naïve group (NS = p > 0.05). Displayed values are mean volume (mm^3^). Error bars represent SEM.

CD8^+^ T cells from the spleens of non tumor-bearing (naïve), IgG2a-pretreated animals (control), or sTGF-βR-pretreated animals (TGF-β-blockade) were mixed with AB12 cells (in a 3:1 ratio of CD8^+^ T cell:tumor cell) and injected into the flanks of different, non tumor-bearing animals. At the time of CD8^+^ T cell isolation, average tumor sizes of the control and TGF-β-blockade groups were 310 and 370 mm^3^, respectively (data not shown). As shown in Figure 4, the mixture of naïve CD8^+^ T cells and AB12 cells resulted in tumors that grew to an average size of approximately 100 mm^3^ after 7 days. This is the same average size as tumors resulting from the inoculation of tumor cells alone (i.e., without CD8^+^ T cells) (data not shown). In comparison, the mixture of control CD8^+^ T cells and AB12 cells resulted in significantly smaller tumors (p <0.05). In contrast, the mixture of TGF-β-blockade CD8^+^ T cells with AB12 cells resulted in tumors that grew to a much larger average size than tumors from the AB12/control CD8^+^ T cell mixture (p < 0.05) and to the same average size as tumors from the AB12/naïve CD8^+^ T cell mixture. These findings demonstrate that the increased rate of AB12 tumor growth after pretreatment with sTGF-βR depends on inhibition of naturally-occurring endogenous anti-tumor CTL activity.

### Pretreatment with sTGF-βR before tumor challenge affects neither the migration of DCs nor their expression of CD86, MHC class I, or MHC class II

We have shown that anti-tumor CTLs develop spontaneously in small AB12 tumor-bearing mice and that these endogenous CTLs are not active when sTGF-βR is given before AB12 tumor cell-inoculation. Anti-tumor CTLs develop from naïve CD8^+^ T cells that are sensitized to tumor antigen when it is presented by antigen-presenting cells (mainly dendritic cells (DCs)) in TDLNs. Initial sensitization of CD8^+^ T cells typically requires 4 steps: migration of DCs into tumor nodules, ingestion and subsequent internal-processing of apoptotic cancer cell debris, presentation of processed peptide fragments in both MHC class I and class II complex clefts (by activated DCs), and migration of the activated DCs into TDLNs where T cell-sensitization occurs. In order to determine if pretreatment with sTGF-βR affects anti-tumor CTLs indirectly through interruption of these 4 steps, we used flow cytometry to study the effect of pretreatment with sTGF-βR on both the number of DCs and the expression of DC-activation markers (CD86, MHC class I and II) in the tumor and TDLNs.

**Figure 5:**
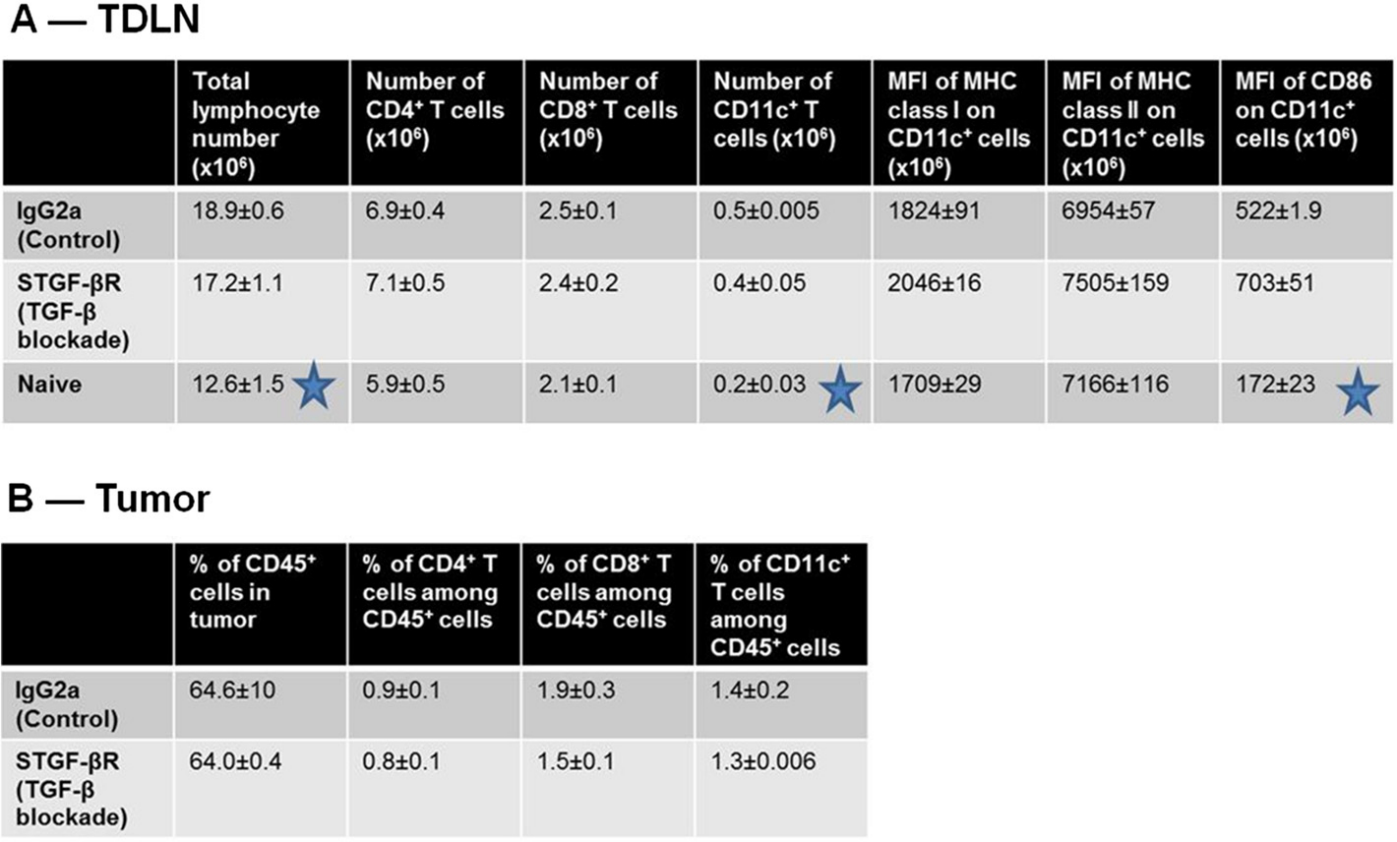
Tumor and tumor-draining lymph node cell counts and mean-fluorescence intensities on day 4 post tumor-inoculation **Tumor and tumor-draining lymph node cell counts and mean-fluorescence intensities on day 4 post tumor-inoculation.** The total number of lymphocytes and DCs in TDLNs (**A**) of tumor-bearing mice were significantly increased compared to naïve non tumor-bearing mice. Furthermore, the expression of CD86 on CD11c^+^ cells was significantly lower in naïve non tumor-bearing mice compared to tumor-bearing mice. However, no significant differences in the total number of DCs, CD8^+^ T cells, or CD4^+^ T cells in TDLNs, or the percentages of CD45^+^, CD4^+^, CD8^+^, or CD11c^+^ in tumors (**B**) were found between tumor-bearing mice pretreated with IgG2a and tumor-bearing mice pretreated with sTGF-βR (* denotes p < 0.05).

The total number of lymphocytes and DCs in TDLNs of mice injected with tumor cells were significantly increased at day 2, 4 and 7 compared to naïve non tumor-bearing mice (Figure 5A). However, no significant differences in the total number of DCs, CD8^+^ T cells, or CD4^+^ T cells in TDLNs were found between tumor-bearing mice pretreated with IgG2a and tumor-bearing mice pretreated with sTGF-βR. Furthermore, no significant differences in the mean fluorescence intensities (MFIs) of CD86, MHC class I, or MHC class II in DCs were found between tumor-bearing mice pretreated with IgG2a and tumor-bearing mice pretreated with sTGF-βR.

When we compared tumors between groups, as expected, the average AB12 tumor weight at day 7 post tumor cell-inoculation in mice pretreated with sTGF-βR was significantly greater than the average tumor size in mice pretreated with IgG2a (data not shown). However, no significant differences were found in the total numbers of tumor-infiltrating CD45^+^ cells, DCs, or CD8^+^ T cells between tumor-bearing mice pretreated with sTGF-βR and tumor-bearing mice pretreated with IgG2a (Figure 5B). These findings demonstrate that the increased rate of AB12 tumor growth resulting from pretreatment with sTGF-βR is not due to an effect on the migration or activation (assessed by expression levels of CD86 and MHC class I and II) of DCs.

### Administration of sTGF-βR to animals with established AB12 tumors does not increase the growth rate of secondary “metastatic” tumors

**Figure 6:**
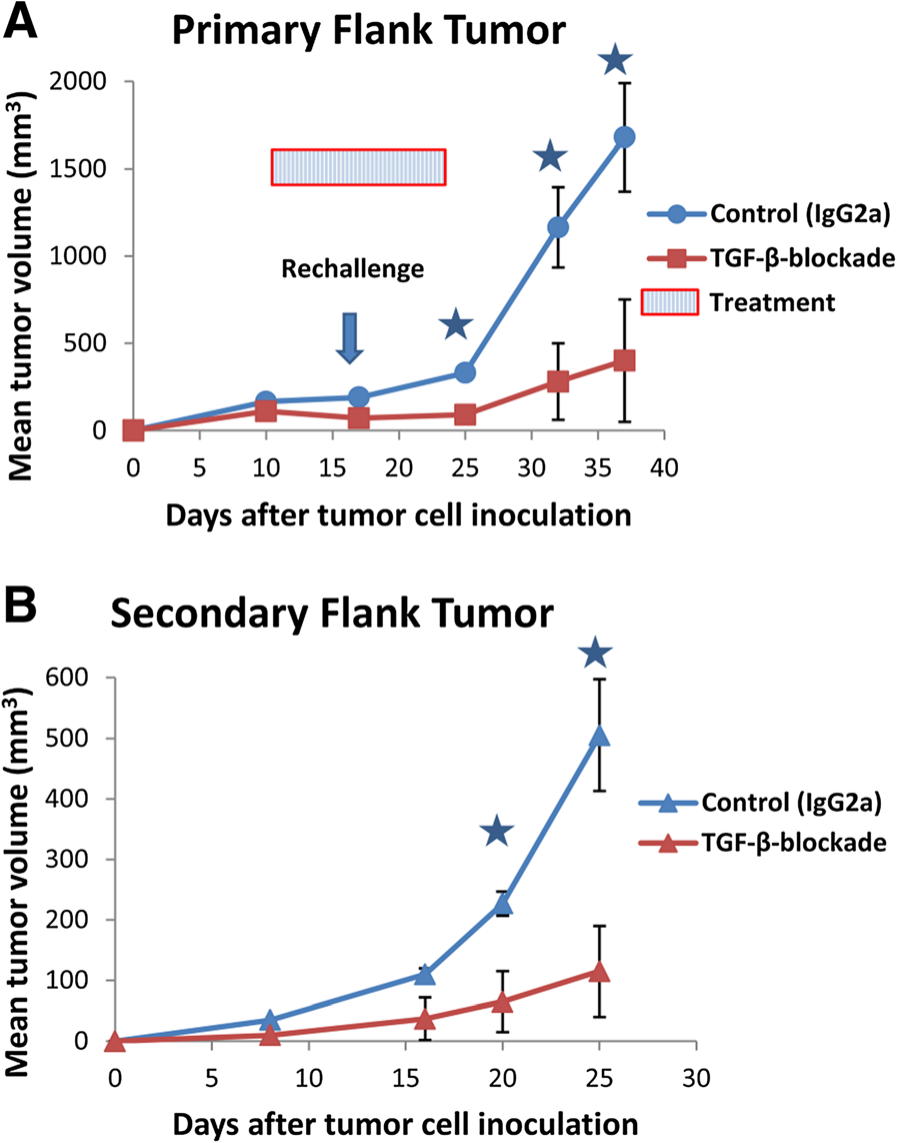
The effect of sTGF-βR on secondary flank tumors in a model of metastatic disease. In all graphs, tumor volume was calculated by the formula (π*long axis*short axis*short axis)/6 and displayed as mean volume (mm^**3**^**).** Error bars represent standard error of the mean (SEM). (**A**). Groups of mice (n=5) bearing small, established AB12 tumors (150 mm^3^) received IP injections of IgG2a (control) or sTGF-βR(TGF-β-blockade), once every 3 days, for a total of 6 doses (marked by hatched bar). After 3 doses of either IgG2a or sTGF-βR, 1x10^6^ AB12 cells were inoculated into the opposite flank to model “metastatic foci”. An arrow shows the time point when secondary tumor cell injection was performed. Tumor volume was subsequently measured over time. Established primary flank tumors in mice treated with sTGF-βR were significantly smaller than tumors in control animals treated with IgG2a at multiple time points (* = p < 0.05). (**B**). The volume (mm^3^) of secondary flank tumors was measured for up to 4 weeks post-inoculation. Secondary tumors in animals treated with sTGF-βR were significantly smaller than secondary tumors in control animals treated with IgG2a at multiple time points (* = p < 0.05).

The inhibition of TGF-β in animals with established tumors reduces tumor growth rates and both augments and preserves anti-tumor CTL function [22]. In contrast, data from the present study suggest that the blockade of TGF-β at the time of tumor initiation inhibits tumor-specific CTLs and augments tumor growth. Given these results, we questioned the therapeutic utility of sTGF-βR in patients who might develop secondary (metastatic) lesions. To determine if the blockade of TGF-β, at a time point after anti-tumor CTLs have been induced, enhances secondary tumor growth, we administered sTGF-βR (TGF-β-blockade) or IgG2a (control) to BALB/c mice after AB12 tumors had formed but before re-challenge with a second AB12 “metastatic focus” in the opposite flank. We then continued treatment with sTGF-βR or IgG2a after the re-challenge and serially measured the volume of both the primary and secondary tumors. As shown in Figure 6A, the administration of sTGF-βR significantly inhibited the growth of small, established AB12 tumors compared to IgG2a (p < 0.05 on Day 25, 32, and 36). Furthermore, the administration of sTGF-βR significantly inhibited the growth of secondary AB12 tumors compared to IgG2a on days 20 (65 mm^3^ vs. 227 mm^3^) and 25 (115 mm^3^ vs. 505 mm^3^) post tumor-inoculation (p < 0.05) (Figure 6B). These results demonstrate that the blockade of TGF-β after anti-tumor CTLs have been induced does not enhance secondary tumor growth.

### Pretreatment with sTGF-βR before immunization with Ad.E7 inhibits the generation of E7-specific CD8^+^ T cells

To determine if TGF-β is required to generate antigen-specific CD8^+^ T cells, we utilized a previously developed adenoviral vector that expresses the well-studied viral tumor antigen human papilloma virus E7 protein (Ad.E7). In this independent and more quantifiable system, we investigated how the blockade of endogenous TGF-β, at a time point before antigen immunization, affected the generation and maintenance of antigen-specific CD8^+^ T cells [32,33].

**Figure 7:**
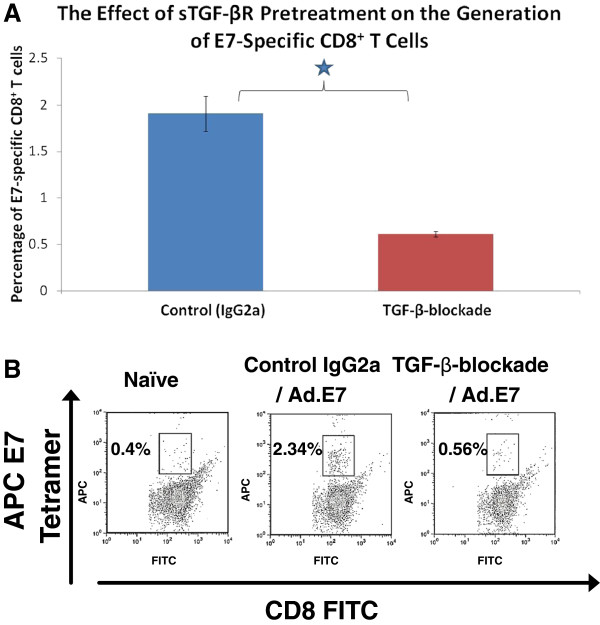
**The effect of pretreatment with sTGF-βR on the generation of E7-specific CD8**^**+ **^**T cells after immunization with Ad.E7. **(**A**). Groups of mice (n=3) were treated with either IgG2a (control) or sTGF-βR 2 days before immunization with Ad.E7. Seven days after immunization, splenocytes from these animals were subjected to flow-cytometric analysis using anti-CD8 antibody and E7 tetramer. Displayed values are mean percentages of E7-tetramer positive CD8^+^ T cells among total splenic CD8^+^ T cells. Error bars represent SEM. The percentage of E7-specific CD8^+^ T cells in animals treated with sTGF-βR (“TGF-β-blockade”) was significantly less than the percentage of E7-specific CD8^+^ T cells in control animals treated with IgG2a (“Control IgG2a”): 0.6% and 1.9%, respectively (* = p < 0.01). (**B**). Examples of two-color (FITC and APC) flow cytometry dot plots in the analysis of CD8^+^ T cells isolated from the groups described in part A. Displayed values are percentages of CD8^+^ E7 tetramer-positive cells among total splenic CD8^+^ T cells. “Naïve” = non-immunized animals; “Control IgG2a/Ad.E7” = animals treated with IgG2a 2 days before immunization with Ad.E7. “TGF-β-blockade/Ad.E7” = animals treated with sTGF-βR 2 days sbefore immunization with Ad.E7.

The average percentage of E7-specific CD8^+^ T cells among total CD8^+^ splenocytes of naïve, non-vaccinated mice is less than 0.5% (see example in Figure 7B). Seven days after immunization with Ad.E7, in control mice pretreated with IgG2a, the average percentage of E7-specific CD8^+^ T cells among total CD8^+^ splenocytes was 1.9% (Figure 7A and example in Figure 7B). In contrast, the average percentage of E7-specific CD8^+^ T cells among total CD8^+^ splenocytes of vaccinated mice pretreated with sTGF-βR was 0.6%, which was significantly lower than the vaccinated control group (p < 0.05) (Figure 7A with an example in Figure 7B). There was no significant difference in the number of splenocytes or percentage of splenocytes that were CD8^+^ between mice pretreated with IgG2a or sTGF-βR. These data suggest that TGF-β is required to generate E7-specific CD8^+^ T cells after immunization with Ad.E7.

### The administration of sTGF-βR after E7-immunization prevents the spontaneous loss of E7-specific CD8^+^ T cells

**Figure 8:**
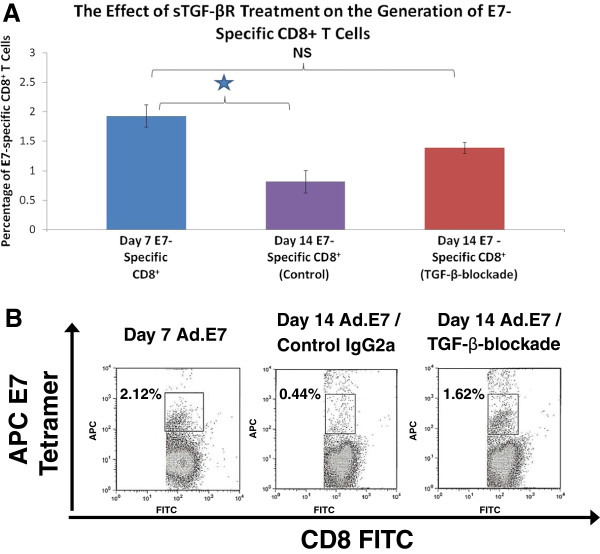
**The effect of treatment with sTGF-βR on established E7-specific CD8**^**+ **^**T cells that were generated by Ad.E7 administration. **(**A**). Groups of mice were immunized with Ad.E7. Seven days post-immunization, splenocytes were isolated and subjected to flow-cytometric analysis (“Day 7 E7-specific CD8^+^ percentage”) using anti-CD8 antibody and E7 tetramer. Displayed values are mean percentages of E7-tetramer positive CD8^+^ T cells among total splenic CD8^+^ T cells. Then, at this time point of 7 days post-immunization, other groups of mice (n=3) were treated with IgG2a or sTGF-βR. At day 14 post-immunization, splenocytes from these animals were isolated and subjected to flow-cytometric analysis. The percentage of E7-specific CD8^+^ T cells from control animals treated with IgG2a on day 14 post-immunization with Ad.E7 (“Day 14 E7-specific CD8^+^, Control IgG2a”) was significantly less than the Day 7 E7-specific CD8^+^ percentage: 0.8% vs. 1.9%, respectively (* = p < 0.05). However, the percentage of E7-specific CD8^+^ T cells from animals treated with sTGF-βR on day 14 post-immunization with Ad.E7 (“Day 14 E7-specific CD8^+^, TGF-β-blockade”) was not significantly different than the Day 7 E7-specific CD8^+^ percentage (NS, p > 0.05). (**B**). Examples of two-color (FITC and APC) flow-cytometry dot plots in the analysis of CD8^+^ T cells isolated from the groups described in part 6A. Displayed values are percentages of CD8^+^ E7 tetramer-positive cells among total splenic CD8^+^ T cells. “Day 7 Ad.E7” = non-treated animals 7 days after immunization with Ad.E7. “Day 14 Ad.E7/Control IgG2a” = animals 14 days after immunization with Ad.E7 and 7 days after IgG2a treatment. Day 14 Ad.E7/TGF-β-blockade = animals 14 days after immunization with Ad.E7 and 7 days after sTGF-βR treatment.

We then utilized the adenoviral vector system to determine if sTGF-βR affects the period of viability of established E7-specific CD8^+^ T cells. Seven days after immunization with Ad.E7, we initiated treatment with either IgG2a or sTGF-βR. At this point in time, before any further intervention, the average percentage of E7-specific CD8^+^ T cells among total CD8^+^ splenocytes was 1.9% (the Day 7 E7-specific CD8^+^ T cell percentage). Seven days after initiating these treatments (14 days after immunization with Ad.E7), this percentage decreased significantly to 0.8% in mice treated with IgG2a (p < 0.05) (Figure 8A) but remained at 1.36% in mice treated with sTGF-βR, a difference which was not statistically different from the Day 7 E7-specific CD8^+^ T cell percentage of 1.9% (p = 0.14) (Figure 8A). Typical flow cytometry plots, after staining for CD8 and E7 tetramer, are provided for each group in Figure 8B. These data suggest that the blockade of endogenous TGF-β, at a time point after immunization with Ad.E7, prevents spontaneous, time-dependent loss of E7-specific CD8^+^ T cells.

## Discussion

Because of its multiple distinct functions in a variety of experimental models of T cell immunology, it has been difficult to develop a clear model of the *in vivo* roles of TGF-β [7,40]. There is ample data to support the hypothesis that TGF-β is an immunosuppressive factor. As summarized previously [3,8,15,16,40], TGF-β has been reported to (1) inhibit T cell proliferation, CTL generation, and T cell cytokine production; (2) interfere withT_H_1/T_H_2 differentiation and the differentiation of naïve T cells towards central memory cells; and (3) inhibit dendritic cell (DC)-mediated antigen presentation by inhibiting DCs’ endocytic and phagocytic activities, preventing DC-maturation, and blocking the up-regulation of critical DC-associated co-stimulatory molecules.

In contrast, there are other studies (although fewer) that have reported that TGF-β exerts stimulatory effects on human T cells and dendritic cells. There is evidence that under some conditions, TGF-β can (1) support the generation of effector cells; (2) augment the development of memory and mature T cell populations; (3) co-stimulate the growth and maturation of CD4^+^ and CD8^+^ T cells; (4) inhibit the apoptosis of CD4^+^ T cells; (5) promote the *in vitro* development of DCs from hematopoietic progenitors; and (6) regulate the chemotaxis of DCs via regulation of chemokine receptor expression [40-46].

Based on the paradigm that TGF-β is “one of the most potent immunosuppressors described to date” [4], translational investigators have tried to inhibit tumor growth in animal models by blocking TGF-β production, receptor binding, or function. Using a number of approaches that include anti-TGF-β antibodies, soluble receptors, or TGF-β-binding proteins [6,17], investigators have consistently reported that blockade of TGF-β is therapeutically useful in a number of murine tumor systems, including renal cell cancer [18], melanoma [19], hepatocellular carcinoma [20], and glioma [21].

The literature is currently unable to bridge these seemingly contradictory findings regarding TGF-β in cancer biology. The observed results likely depend on the experimental models used, the type of stimulus, the presence of other cytokines, the dose of TGF-β, the distribution of TGF-β in its latent and active form, the duration of the stimulation, and possibly, the genetic background of the cell populations studied [40]. Regardless of the reasons, since TGF-β blocking agents are currently being developed for clinical use, it has become increasingly important to better understand the effects of TGF-β on *in vivo* anti-tumor immune cell function.

We observed that blockade of TGF-β with sTGF-βR before the inoculation of tumor cells (and for 3 doses afterwards) resulted in significantly enhanced tumor growth of one particular tumor cell line, the AB12 line (but not in others). This response was in marked contrast to the inhibition of tumor growth associated with administration of the same TGF-β blocking agent after the establishment of the same tumor cell line. In this study, we examined the mechanism responsible for the increased rate of AB12 tumor growth resulting from pretreatment with sTGF-βR. We demonstrated that altered anti-tumor immune responses were responsible for this augmentation of tumor growth; specifically, administration of sTGF-βR before tumor cell-inoculation resulted in the failure to generate active anti-tumor CTLs.

The specific characteristics of the relatively immunogenic tumor model used in these studies are important to understand our findings. Mesotheliomas usually result from prior asbestos exposure. They are associated with a high degree of MHC class I expression and TGF-β production. Clinically, they respond to some immune-based therapies. The mouse mesothelioma tumor cells used in this study are very similar to human tumors. When AB12 cells are injected into syngeneic BALB/c mice, their initial growth is quite slow until about 20 days, at which point their size begins to increase rapidly (Figures 2A, 2E, and 6A). It appears that this initial slow growth phase is due to a partially effective anti-tumor immune response mediated by endogenous, functionally active tumor antigen-specific CTLs. We have observed that AB12 tumors grow much more rapidly in SCID mice (Figure 2F), in CD8^+^ T cell-depleted mice (Figure 3B), and in IFNγ-knockout or IFNγ-neutralized mice (data not shown). We have also directly examined the ability of AB12 tumors to generate anti-tumor immune responses. Within 4–10 days after subcutaneous injection of AB12 tumor cells, we have detected CD8^+^ T cells in the spleen that have cytolytic activity [22]. We confirmed the presence of these spontaneously generated anti-tumor CTLs in this study (Figure 4) using a Winn assay that demonstrated markedly inhibited tumor growth when tumor cells were mixed with CD8^+^ splenocytes from control tumor-bearing animals before inoculation into naïve non tumor-bearing animals. These anti-tumor CTLs persist until the tumor reaches a size of approximately 400 mm^3^ (usually about 20 days after injection). At this time, CTL activity can no longer be detected and tumor growth rate rapidly increases.

Our experiments indicate that the increased rate of AB12 tumor growth resulting from pretreatment with sTGF-βR was due to a loss of this normal, low-level, and only partially-effective anti-tumor CTL immune response. First, the growth-augmenting effects of sTGF-βR relative to IgG2a were lost in T cell-deficient SCID mice (Figure 2F) and CD8^+^ T cell-depleted mice (Figure 3B). Second, we showed that the inhibition of TGF-β negatively impacts the functionality of CD8^+^ CTLs, as the Winn assay (essentially an *in vivo* test of CD8^+^ T cell functionality) demonstrated a reduced anti-tumor response with an equivalent number of CD8^+^ T cells from mice pretreated with sTGF-βR compared to control animals pretreated with IgG2a (Figure 4). Together, these results implicate the inhibition of anti-tumor CD8^+^ CTLs as central to the augmentation of AB12 tumor growth associated with sTGF-βR pretreatment.

In addition to our tumor study, we also investigated the effect of TGF-β-blockade on the generation of active antigen-specific CTLs against a known viral tumor antigen in an independent and more quantifiable system. Pretreatment with sTGF-βR, at a time point before immunization with an adenovirus encoding the HPV E7 protein (Ad.E7), inhibited the generation of E7-specific CD8^+^ T cells as compared to control pretreatment with murine IgG2a. These experiments show that TGF-β is required for the generation of active CTLs, at least in models employing AB12 tumor cells or vaccination with Ad.E7.

Unfortunately, despite further investigation, the mechanism by which pretreatment with sTGF-βR inhibits CTL-activity remains unclear. Initial sensitization of CD8^+^ T cells typically requires 4 steps as described above. We showed that pretreatment with sTGF-βR does not decrease the activation status or the number of DCs, CD4^+^ T cells, or CD8^+^ T cells in the TDLNs or tumor beds compared to IgG2a. These data indicate that TGF-β may not be required for the migration or proliferation of DCs, CD4^+^ T cells, or CD8^+^ T cells or the activation of DCs. Although studies of expression levels of CD86, MHC class I, and MHC class II are important to evaluate the activation levels of DCs in anti-tumor immune responses, other activation markers for DCs might exist, such as ICAM-1 or B7. It may also be important to test the expression levels of accessory molecules on T lymphocytes, such as LFA-1 or CD28. Thus, the mechanism by which pretreatment with sTGF-βR stimulates the growth of tumors in our AB12 tumor model remains unclear.

Another interesting question relates to the issue of why sTGF-βR did not inhibit the generation of anti-tumor CD8^+^ CTL activity in other tumor models as it did in the AB12 tumor model. We explored a number of obvious explanations: low amounts of TGF-β produced, lack of tumor immunogenicity, or animal strain differences. With regard to TGF-β production, we know that AB-1 cells make very little TGF β which could explain the lack of effect in this cell line. However, the TC-1 cell line makes sizeable amounts of TGF-β and yet it is still resistant. We have also studied the L1C2 and TC-1 cell lines in the past and have shown them to be moderately or highly immunogenic, similar to the AB12 model, and able to induce anti-tumor CD8^+^ T cells [47]. To address the issue of strain differences, we also studied L1C2 cells, another tumor line that grows in BALB/c mice (like AB12), and saw no response. We thus have no simple explanation for the selectivity for our observation. The tumor microenvironment is a complex ecosystem which is unique to each tumor model. Given the genetic modifications required for malignant transformation, it is likely that a myriad of factors, including various cytokines, chemokines, other soluble factors, and even cell-bound mediators play significant roles in tumor development and in the interaction with the host’s immune system. The key point is that this stimulation of tumor growth after early TGF-β inhibition can occur in at least one animal model and thus should be carefully looked for in future clinical trials. Additional ongoing research that identifies the key factors responsible for this effect will be needed.

## Conclusions

In conclusion, this paper provides the first *in vivo* evidence, to our knowledge, that the blockade of TGF-β inhibits the initial generation of functionally active anti-tumor CTLs and antigen-specific CD8^+^ T cells after Ad.E7 vaccination. These findings support the novel hypothesis that, at least under some circumstances, TGF-β is required for the generation of active anti-tumor CTLs. Given the complexity of the *in vivo* anti-tumor immune response, we have not yet defined the step at which TGF-β-blockade inhibited CTL activation. Although pretreatment with sTGF-βR may not be involved in the migration of immune cells (Figure 5), possible mechanisms include inhibition of either antigen presentation by DCs or other antigen-presenting cells, T cell differentiation, or generation of memory/effector cells. Experiments to differentiate among these potential mechanisms are in progress.

The implications of our findings are significant. From an immunological standpoint, our results support the complex *in vivo* functions of TGF-β and suggest a potentially new paradigm for its role in the generation of CD8^+^ memory and/or effector cells. Since it is extremely difficult to model all the variables that factor into an *in vivo* immune response, it will be very important to study the effects of TGF-β manipulation in a variety of animal models. From a more practical standpoint, these results may help guide the use of TGF-β inhibitors. Given our observation that TGF-β is required for anti-tumor immune responses, along with other data showing that TGF-β-blockade can enhance carcinogenesis through tumor cell-intrinsic mechanisms [48-50], the use of TGF-β inhibitors in a chemopreventive mode should be undertaken with caution. On the other hand, the use of TGF-β inhibitors in patients with established tumors might prove very useful. One encouraging finding from our study was that the blockade of TGF-β did not lead to increased growth rates at secondary (“metastatic”) sites. These data support the hypothesis that blockade of TGF-β does not enhance tumor growth after anti-tumor CTLs have been induced.

We also have evidence from the Ad.E7 model that TGF-β-blockade promotes the persistence of established antigen-specific CD8^+^ T cells that were induced by immunization at a time point prior to sTGF-βR administration (Figure 8B). While the percentage of E7-specific CD8^+^ T cells in control animals decreased significantly 1 week after IgG2a administration, the percentage of E7-specific CD8^+^ T cells in animals treated with sTGF-βR remained stable at the same time point. These results thus support the use of TGF-β-inhibition in patients with established tumors.

In summary, we present an *in vivo* tumor model demonstrating that the timing of TGF-β blockade can determine whether tumor growth is inhibited or enhanced. These experiments highlight the pleomorphic effects of TGF-β and emphasize the importance of careful patient-selection for novel TGF-β inhibitors.

## Abbreviations

TGF-β: Transforming growth factor-β; sTGF-βR: Soluble Type II TGF-β receptor; IP: Intraperitoneal; FACS: Fluorescence-activated cell sorting; SC: Subcutaneously; SCID: Severe combined immunodeficiency; ANOVA: Analysis of variance; CTLs: Cytotoxic CD8+ T cells; DC: Dendritic cell; Ad.E7: Adenoviral vector encoding the human papillomavirus E7 gene; MLECs: Non-malignant mink lung epithelial cells; PAI-1: Plasminogen activator inhibitor-1; FBS: Fetal bovine serum; DMEM: Dulbecco’s modified Eagle’s medium; RLU: Relative light units; TILs: Tumor-infiltrating lymphocytes; TDLNs: Tumor-draining lymph nodes; APC: Allophycocyanin; FITC: Fluorescein isothiocyanate; PE: Phycoerythrin; pfu: Plaque-forming units; MHC: Major histocompatibility complex; MFI: Mean fluorescence intensity.

## Competing interests

The authors report no editorial or financial conflict of interest.

## Authors’ contributions

SA, SS, ES conceived of the study. Data acquisition performed by ES, BJ, OO, PB, OV, EE, JP. Data analysis performed by ES, SA. Reagents contributed by SA, SS. Manuscript preparation performed by JQ, ES, SA, SS. Manuscript finalization and submission performed by JQ. All authors read and approved the final manuscript.
